# Viscoelastic behaviour of human mesenchymal stem cells

**DOI:** 10.1186/1471-2121-9-40

**Published:** 2008-07-22

**Authors:** Samuel CW Tan, Wen X Pan, Gang Ma, Ning Cai, Kam W Leong, Kin Liao

**Affiliations:** 1 Division of Bioengineering, School of Chemical and Biomedical Engineering, Nanyang Technological University, Singapore 637457, Singapore; 2 Biomedical Engineering Research Center, School of Electrical & Electronic Engineering, Nanyang Technological University, Singapore 637553, Singapore; 3 Duke-National University of Singapore Graduate Medical School, Singapore 169547, Singapore; 4 Department of Biomedical Engineering, Duke University, Durham, NC 27708, USA

## Abstract

**Background:**

In this study, we have investigated the viscoelastic behaviour of individual human adult bone marrow-derived mesenchymal stem cells (hMSCs) and the role of F-actin filaments in maintaining these properties, using micropipette aspiration technique together with a standard linear viscoelastic solid model.

**Results:**

Under a room temperature of 20°C, the instantaneous and equilibrium Young's modulus, *E*_0 _and *E*_∞_, were found to be 886 ± 289 Pa and 372 ± 125 Pa, respectively, while the apparent viscosity, *μ*, was 2710 ± 1630 Pa·s. hMSCs treated with cytochalasin D up to 20 μM at 20°C registered significant drop of up to 84% in stiffness and increase of up to 255% in viscosity. At the physiological temperature of 37°C, *E*_0 _and *E*_∞ _have decreased by 42–66% whereas *μ *has increased by 95%, compared to the control. Majority of the hMSCs behave as viscoelastic solid with a rapid initial increase in aspiration length and it gradually levels out with time. Three other types of non-typical viscoelastic behavior of hMSCs were also seen.

**Conclusion:**

hMSCs behave as viscoelastic solid. Its viscoelstic behaviour are dependent on the structural integrity of the F-actin filaments and temperature.

## Background

Mesenchymal stem cells (MSCs), a type of the adult stem cells that can be harvested from bone marrow and other sources such as liver, umbilical cord, placenta, adipose tissue, synovial membrane, amniotic fluid and even teeth [1], have increasingly played a central role in regenerative medicine. Their attractiveness is found in their multipotency to differentiate and develop into various types of tissues such as adipose, cartilage and bone [2], as well as their promising use in patient-specific gene therapy [1].

Similar to other cell types, the viability and function of hMSC are influenced by their microenvironment, including the presence of mechanical stimuli. One pathway by which mechanical stimuli may alter gene expression is through a direct physical connection from the extracellular matrix across the membrane and cytoplasm to the interconnected network of cytoskeleton, and eventually reaches the nucleus [3,4]. Many studies, both *in vivo *[5-7] and *in vitro *[8-13], have demonstrated that external mechanical stimuli can induce MSCs differentiation into various lineages. More recently, it has been shown that stem cell lineage specification is directed by matrix elasticity [14], and that rheological character of stem cell nucleus is closely related to differentiation [15]. Because cell fate is influenced by these interacting elastic/viscoelastic bodies (cell and the substrate) in a dynamic way, it is imperative to accurately measure the mechanical properties of the stem cells. These fundamental properties are needed in future studies in understanding, for instance, how mechanical stimuli of various forms, may influence the fate of stem cells. Such information is also required in setting foundation for any future studies correlating mechanical properties with the state of differentiation of stem cells, and in modeling stem cell behaviour in a systematic way.

As a first step towards a better understanding of the role of mechanotransduction in MSC differentiation, this study aimed to study the viscoelastic behaviour of hMSCs and to obtain baseline data, as they are still scarce to date [16]. Micropipette aspiration, a well-established technique [17-19], was used to study and quantify the mechanical properties of hMSCs. From a mechanics and materials point of view, the viscoelastic behaviour of materials depends on material structure and temperature. As such, the role of F-actin, one of the components of cytoskeleton, and the temperature effect on the mechanical properties of hMSCs were investigated by adding cytochalasin D, a chemical agent that disrupts actin, and by examining the cells at 20°C (a convenient "room temperature" at which most material testing were performed, we thus arbitrarily named data obtain at this temperature "control") and 37°C, the physiological temperature, respectively. Such data will provide starting point for quantitative, systematic description and modelling of stem cell behavior.

## Results

### Viscoelastic behaviour of hMSCs

**Figure 1:**
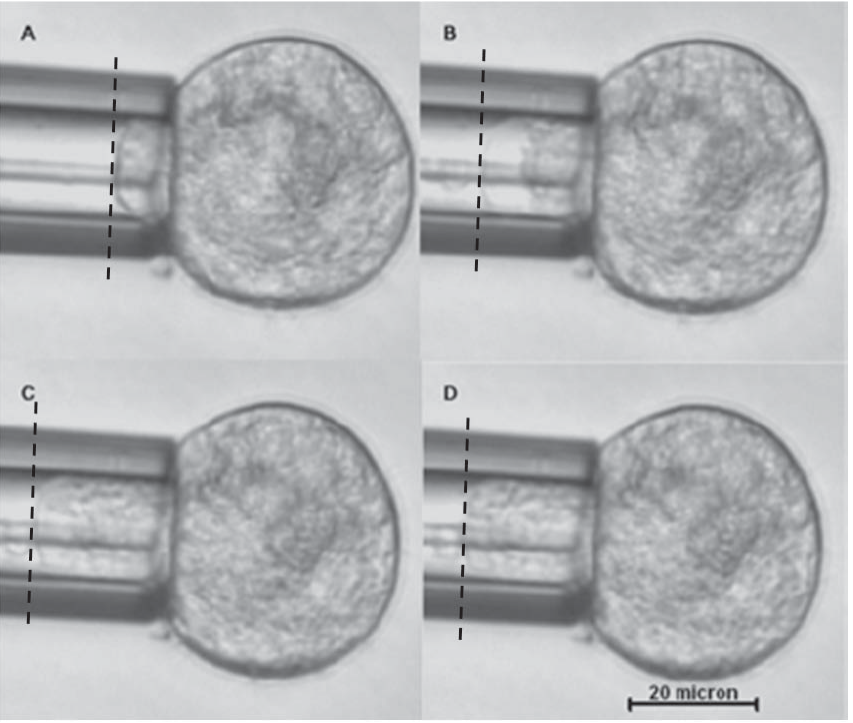
**Micropipette aspiration of hMSCs at room temperature**. Images (A-D) are displayed at time t = 1 s, 15 s, 100 s and 200 s after the application of step aspiration pressure, respectively. An initial jump of cell protrusion (A) into the micropipette in response to a step aspiration pressure is followed by an asymptotic creep behavior and eventually reaching equilibrium.

**Figure 2:**
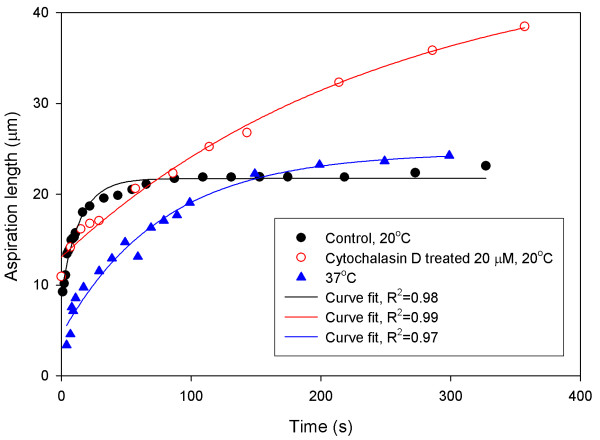
**Various patterns of aspiration length at different conditions with respect to time**. hMSCs treated with 20 μM of cytochalasin D at 20°C achieve the longest aspiration length. hMSCs reach the equilibrium aspiration length faster at 20°C than 37°C.

**Figure 3:**
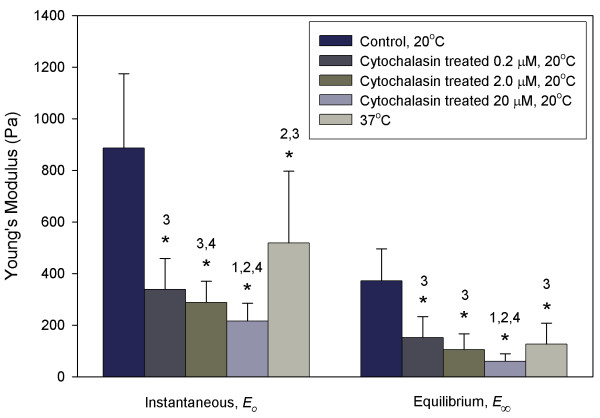
**Effects of temperature and cytochalasin D on the instantaneous and equilibrium Young's modulus of hMSCs**. The stiffness of hMSCs decreases with increasing temperature and increasing concentration of cytochalasin D. *p < 0.001 as compared to control. (1) *p *< 0.05 as compared to cytochalasin treatment at 0.2 μM. (2) *p *< 0.05 as compared to cytochalasin treatment at 2.0 μM. (3) *p *< 0.05 as compared to cytochalasin treatment at 20 μM. (4) *p *< 0.05 as compared to cells at 37°C.

**Figure 4:**
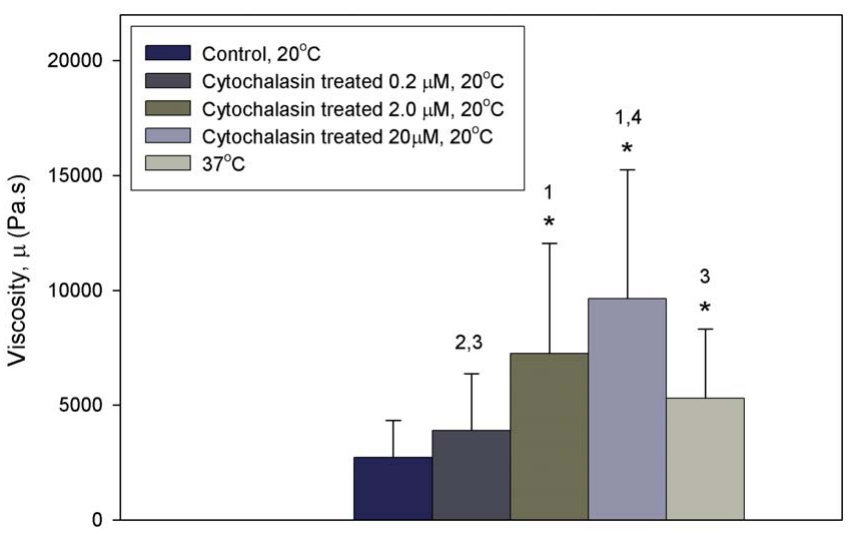
**Dependence of viscosity of hMSCs on temperature and the integrity of actin cytoskeleton**. *p < 0.05 as compared to control. (1) *p *< 0.05 as compared to cytochalasin treatment at 0.2 μM. (2) *p *< 0.05 as compared to cytochalasin treatment at 2.0 μM. (3) *p *< 0.05 as compared to cytochalasin treatment at 20 μM. (4) *p *< 0.05 as compared to cells at 37°C.

Upon the application of a step pressure to a hMSC at the control temperature of 20°C, the aspiration length showed an initial jump, followed by a gradual increase, and eventually reached its equilibrium length after 100 s (Fig. 1). This observation demonstrated that hMSCs exhibit a typical monotonic viscoelastic behaviour as do many other engineering materials, which was confirmed by a very high mean correlation coefficient of R^2 ^= 0.98 when the aspiration length-time data were fitted with a viscoelastic solid model (Fig. 2). The instantaneous Young's modulus, *E*_0_, the equilibrium Young's modulus, *E*_∞_, and the apparent viscosity, *μ*, of hMSCs obtained were: 886 ± 289 Pa, 372 ± 125 Pa and 2700 ± 1600 Pa·s, respectively (Figs. 3 and 4).

### Effect of Disruption of F-actin

**Figure 5:**
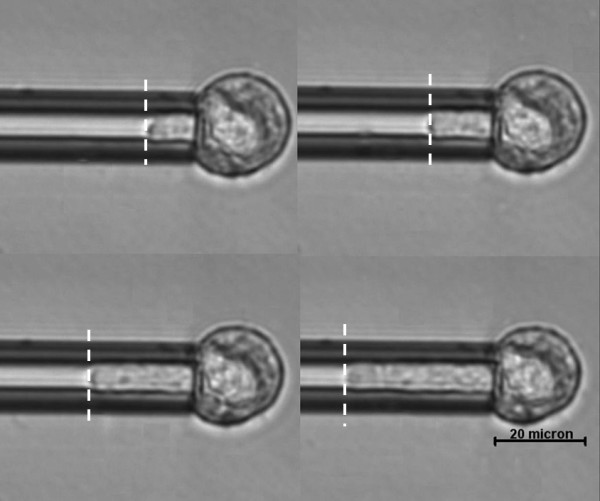
**Micropipette aspiration of hMSCs treated with the highest concentration (20 μM) of cytochalasin D (A – D)**. Images are displayed at T = 1 s, 15 s, 100 s and 200 s after the application of step aspiration pressure, respectively. The aspiration length of the cell increased significantly due to the disruption of F-actin filaments.

The F-actin network of hMSCs was disrupted to certain degree after treatment with cytochalasin D at 20°C (Fig. 5). Under such condition, the hMSCs no longer displayed the typical viscoelastic behaviour of solid materials. The cells experienced a much slower rate of increase in aspiration length with no obvious initial jump, and tended towards a much higher equilibrium length compared to cells tested under the control condition (Fig. 2). The addition of cytochalasin D at various concentrations caused *E*_0 _and *E*_∞ _to decrease significantly by 67% to 84% from the control (*p *< 0.001) (Fig. 3). There was no significant change in *E*_0 _and *E*_∞ _when the concentration was increased from 0.2 μM to 2.0 μM, the IC_50 _value for hMSC (*p *> 0.1). The apparent viscosity, *μ*, increased significantly from the control condition and low concentration of cytochalasin D (0.2 μM) by up to 255% as the concentration was increased to 2.0 μM and 20 μM (*p *< 0.05) (Fig. 4).

### Temperature effect on the viscoelastic behaviour of hMSCs

hMSCs aspirated at 37°C also exhibited typical viscoelastic behaviour. However, the initial jump of the aspirated length was much shorter than the cells tested at 20°C (Fig. 2). The rate of increment of aspiration length decreased gradually with time before it reached the equilibrium aspiration length, which was longer than those tested at 20°C, but lower than those treated with cytochalasin D. Both *E*_0 _and *E*_∞ _of hMSCs determined at 37°C were significantly reduced from the control, to 518 ± 280 Pa and 126 ± 81 Pa, respectively (*p *< 0.001) (Fig. 3), which represent a corresponding reduction of 42% and 66%. The apparent viscosity, *μ*, increased significantly from the control by 95% to 5290 ± 3026 Pa·s (*p *< 0.05) (Fig. 4).

**Figure 6:**
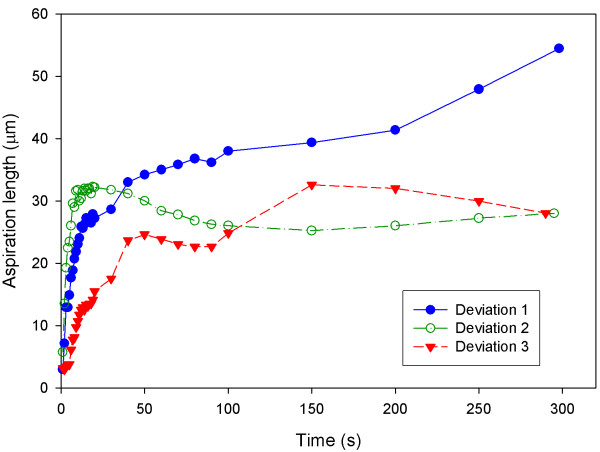
**Deviation patterns of non-typical viscoelastic creep response of hMSCs**. After reaching equilibrium length, 34% of the hMSCs experienced a further increase of length with time (deviation 1) while 14% of the hMSCs reached a peak aspiration length before dropping to a lower equilibrium length (deviation 2). A small amount of hMSCs (7%) exhibited a step-wise deformation behavior (deviation 3). Lines connecting data point indicate data trend.

During the experiment, three other types of viscoelastic behaviour of hMSCs that constitute about 55% of total observations, represented by the aspiration length-time curves, were observed (Fig. 6). Instead of reaching a stable equilibrium aspiration length, close to 34% of the hMSCs displayed further increase of aspiration length with time (deviation 1). 14% of the hMSCs reached maximum aspiration length before decreased to a lower equilibrium length (deviation 2). A small amount of hMSCs (7%) demonstrated a step-wise viscoelastic behaviour in the aspiration length-time curve (deviation 3), where the first step of aspiration length increment occurred at t = 50s while the second step at t = 150s.

## Discussion

A liquid-like cell with a constant cortical tension will flow easily into a micropipette when the suction pressure exceeds the critical pressure [18]. On the other hand, a solid-like cell will reach an equilibrium length of projection instead [18]. Based on previouse studies on other cell types and the results obtained in this study, it can be concluded that hMSCs possess the charateristics of solid-like cells. The calculated equilibrium Young's modulus of hMSCs in this study falls in the same order of 0.5 kPa, compared to porcine endothelial cells and human chrondocytes, which are treated as as viscoelastic solid material (Table 1).

**Table 1:** The comparison of mechanical properties obtained for various cells

Cell types	Mechanical properties			References
		
	*E*_0 _(Pa)	*E*_∞ _(Pa)	*μ *(Pa·s)	
Porcine endothelial	429	114 ± 35	8300 ± 4000	[19]
Human chondrocyte	640	270	2100	[17]
hMSC	886 ± 289	372 ± 125	2700 ± 1600	present work

Both temperature and disruption of F-actin affected the viscoelastic behviour of hMSCs. The increase in viscosity of these cells is attributed to changes in the mechanical integrity of the cell body spanned by the cytoskeleton. Microtubule, one of the major components of cytoskeleton that provides support for compressive load on cells [20], are known to be temperature-sensitive: a decrease in temperature suppresses structure instability by slowing down the rate of catastrophe or shrinkage [21,22]. However, lowering temperature may also imply a reduction in the net amount of assembled microtubules. That is to say, higher temperature promotes microtubule instability as more of them are forming network. From the results obtained, it seems that the former is dominating the two competing effects, which may explain the drop in cell stiffness when the experiments were performed at 37°C, compared to the data at 20°C. Further clarification regarding the two competing effects is needed, however, in future studies.

As expected, the disruption of F-actin by cytochalasin D has the highest impact on the stiffness of hMSCs, leading to the lowest Young's modulus registered. Actin, the major protein components in eukaryotic cells, plays an important role in cell motility and in maintaining cell shape [23]. For suspended cells, actin filaments are found to concentrate on the cell periphery or cortex [24]. Cytochalasin D degrades the actin network into shorter filaments in a dose-dependent manner [24]. As a result, the reduced density of the cortical actin network renders the cell softer, more viscous, and more liquid-like, leading to a higher equilibrium aspiration length (Fig. 2).

Interestingly, three types of deviations from the typical viscoelastic behaviour were observed during the study (Fig. 6). The first deviation (deviation 1) involves a further increment of aspiration length after reaching a seemingly equilibrium state, which could be attributed to mechanical disruption of the cytoskeleton towards the later stage of the aspiration process, causing the cell to turn liquid-like. This kind of behaviour is indeed very similar to the so-called tertiary state in creep behaviour of materials, when a material elongates abruptly due to internal damage and eventually ruptures.

The second deviation (deviation 2), which is characterized by a peak before reaching the equilibrium length, may arise due to either global or localized cell contraction, by rearrangement of actin cytoskeleton after reaching the maximum aspiration length. The third deviation (deviation 3) with a step-wise pattern could be interpreted as the result of a combination of large cell deformation and contraction during aspiration. These patterns, however, were not observed in the hMSCs treated with cytochalasin D, suggesting that the disruption of actin directly affects the deformation behaviour of cells. The results obtained from these three deviations were excluded in the calculation of Young's modulus and viscosity. It should be mentioned that the mechanisms for the latter two deviations are unclear at present. Further experiments with larger pressure range (pressure range for the present study is 500–1000 Pa) may be desirable in order to show how aspiration length-time curves respond to a range of pressure and subsequently constructing possible mechanisms.

Experiments invloving F-actin demonstrated the importance of cytoskeleton in controlling the viscoelastic characteristics of hMSCs. Systematic disruption of other cytoskeletal components such as microtubules and intermediate filaments using relevant chemicals will be performed in subsequent studies, to provide a better understanding of the roles of each of the cytoskeletal components in relation to cell deformation and mechanical properties. Work is also underway to perform live cell staining using green flouoroscence protein (GFP) tag to establish the relationship between the deformation at cellular and cytoskeleton levels. It should be mentioned that the spatial location of nucleus may contribut to the overall deformation of a cell in an aspiration experiemnt and thus influence the aspiration length-time curve. For instance, if the stiffer nucleus is near the tip of the micropipette, it could be anticipated that the aspiration length would be shorter compared to the case where the nucleus is away from the tip. However, quantiative and systemaic analyses of the effect of nucleus can only be revealed by compuational methods.

## Conclusion

In summary, our findings validated the hypothesis that hMSCs behave like a viscoelastic solid material, consistent with other cell types such as chrondocytes and endothelial cells. In addition to typical viscoelastic behviour, three other types of non-typical viscoelastic behaviours of hMSCs were also seen. We have also demonstraed that both instantaneous and equilibrium Young's modulus, as well as the apparent viscosity of hMSCs were sensitive to temperature and the structural integrity of F-actin filaments. The baseline data for hMSCs obtained in this study is expected to facilitate future studies in understanding the relationship between mechanical stimuli and phenotypic alterations of hMSCs, and quantitative description of hMSC behaviour.

## Methods

### Culture of hMSCs and disruption of microfilaments

The hMSC cell line, derived from human bone marrow, was purchased from Cambrex Bio Science Walkersville, Inc., USA. hMSCs were cultured at 5% CO_2_, 37°C using MSC growth medium (MSCGM) (Cambrex, USA), which contains the mixture of MSC basal medium (MSCBM) and supplements such as MSC Growth Supplement (MCGS), L-glutamine, and penicillin/streptomycin. The cells were cultured for 5 days to about 70–80% confluent before harvesting. Once reached 80% confluence, the hMSCs were harvested with 0.25% Trypsin/EDTA (Invitrogen, USA), applied for 5 minutes. After adding appropriate amount of stem cell medium to neutralize trypsin, the suspended cells are given 1 minute to recover before they are plated on a coverslip (0.17 mm thick) for micropipette aspiration experiments.

hMSCs used in the study did not exceed passage number 9 as hMSCs may lose its multi-lineage potential at a later passage number [25]. Cytochalasin D (Sigma, USA) was added to the cell culture at three different concentrations (0.2 μM, 2.0 μM and 20 μM) and incubated for 3 hours prior to detachment by trypsin [24,26]. The number of cells analysed for the respective experimental conditions is shown in Table 2.

**Table 2:** The corresponding cell number of hMSCs under the different experimental conditions

Treatment	Concentration of cytochalasin-D, (*μ*M)	Cell number, *n*
20°C (control)	0	24
Low	0.2	11
Medium	2.0	13
High	20.0	17
37°C	0	13

### Micropipette aspiration

Micropipette aspiration technique was deployed to study the viscoelastic behaviour of hMSCs by applying a suction pressure on the surface of a cell, which was suspended in hMSC growth medium and deposited on a glass coverslip. A heating stage (Heatable Universal Mounting Frame M-H (Zeiss) connected to a temperature regulator, Tempcontrol 37-2 digital (Zeiss)) was used for the experiment conducted at 37°C. Micropipettes were fabricated and refined by drawing borosilicate glass capillary tubes (1.0 mm outer diameter, 0.5 mm inner diameter, Sutter Instrument Co., USA.) using a horizontal pipette puller (Flaming/Brown micropipette puller, model P – 97, Sutter Instrument Co., USA.) and a microforge (Narishige, Japan), respectively, to obtain a range of inner diameter from 7 to 11 μm, which would maintain a constant cell – diameter to inner – micropipette – diameter ratio. Micropipettes were coated with Sigmacote chemical (Sigma, USA) to prevent cell adhesion on the wall of the micropipettes during aspiration.

Before starting an experiment, air bubbles were removed from the tubing by fluid flow. The micropipette was filled with distilled water through a flexible needle attached to a syringe and placed into the micropipette holder. The suction pressure and the position of the micropipette were controlled by a micromanipulator (Narishige, Japan). The amount of pressure exerted on the cells was measured by an in-line pressure transducer (Model DP15 – 30, Validyne Engineering Corp., USA) with a resolution of 1 Pa and was converted to voltage reading using a demodulator (Model CD280-2C, Validyne Engineering Corp., USA) and a digital multimeter (Model 115, Fluke, USA). Each experimental session was limited to within 1 h.

The initial diameter of cells and the equilibrium pressure, at which point the cells were neither aspirated nor pushed away, were obtained before the start of each aspiration to minimize the error incurred by any drift to the measurement of the pressure. A constant step pressure, Δ*P*, ranging from 500 to 1000 Pa, was then applied and the aspiration process was recorded for 350 s using a brightfield microscope (Zeiss Axiovert S100, Germany) equipped with a CCD camera (RT Monochrome, Diagnostic Instruments Inc., USA) through a 40× objective lens (Carl Zeiss, Germany). The length of the cell aspirated into the micropipette was measured using a line tool provided by SPOT software (Diagnostic Instruments Inc., USA) at regular interval and with an accuracy of ± 0.3 μm.

### Theoretical modelling

**Figure 7:**
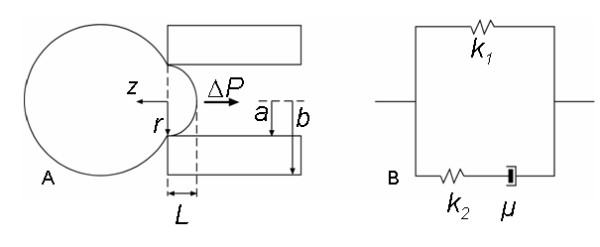
**Schematics of theoretical model of the micropipette aspiration test (Adapted from **[17]**)**. (A) A model cell is being sucked, under a pressure *ΔP*, into a micropipette with inner and outer radius *a *and *b*, respectively. The portion of the cell inside the micropipette is *L. r and z *are coordinates fixed on the cell at the tip of the micropipette. (B) Viscoelastic model of the cell: a spring with elastic constant *k*_1 _is connected in parallel with a spring (elastic constant *k*_2_) and a dashpot (apparent viscosity μ) connected in series.

A linear, three-parameter viscoelastic solid model was used to determine the mechanical properties of hMSCs [19,27], assuming that the pressure applied on cell is uniform and axisymmetric with a boundary condition of no axial displacement of the cell at the micropipette opening. A schematic of the model is shown in Fig. 7. The cell is also assumed to have intrinsic incompressibility and isotropy (Poisson's ratio = 0.5) [19]. The displacement of cell into the micropipette as a function of time, *L(t)*, is

(1) $$L(t)=\frac{\Phi a\Delta P}{\pi k_{1}}\left[1-\frac{k_{2}}{k_{1}+k_{2}}e^{-t/\tau }\right]$$

where Φ is the wall function related to the ratio of the micropipette wall thickness to the pipette radius. In this study, which is based on the *punch *model [27], Φ = 2.0 for the entire range of micropipette inner and outer diameter used. Inner radius of the micropipette and the applied aspiration pressure are represented by *a *and Δ*P*, respectively. The critical pressure,Δ*P*_*c*_, occurs at *L*/*a *= 1. That is, when the cell shape within the micropipette is hemispherical [18]. The apparent viscosity, *μ*, is given by [19]

(2) $$\mu =\frac{\tau k_{1}k_{2}}{k_{1}+k_{2}}$$

where *τ *is the exponential time constant while *k*_1 _and *k*_2 _are the elastic constants which can be determined by solving Eq. (2) using nonlinear regression. Both constants are related to standard elasticity coefficients by the following equations:

(3) $$\begin{array}{cc} E_{0}=\frac{3}{2}\left(k_{1}+k_{2}\right), & E_{\infty }=\frac{3}{2}k_{1} \end{array}$$

where *E*_0 _is the instantaneous Young's modulus and *E*_∞ _is the equilibrium Young's modulus [17].

Statistical analyses were performed using SigmaPlot version 8.02 software (SYSTAT Software, Inc., USA). Unpaired two-sample (with unequal variance) *t*-test was used to examine whether the means calculated are significantly different.

## Authors' contributions

SCWT and WXP carried out experimental studies, data collection, statistical analysis, and drafted the manuscript, they contributed equally to the present work. GM participated in the experimental setup and data collection while NC prepared cells. KWL and KL participated in the design and coordination of the study, as well as critical reading of the manuscript. All authors read and approved the final manuscript.
